# A modified Delphi study to develop a practical guide for selecting patients with prostate cancer for active surveillance

**DOI:** 10.1186/s12894-021-00789-5

**Published:** 2021-02-04

**Authors:** Samuel W. D. Merriel, Daniel Moon, Phil Dundee, Niall Corcoran, Peter Carroll, Alan Partin, Joseph A. Smith, Freddie Hamdy, Caroline Moore, Piet Ost, Tony Costello

**Affiliations:** 1 College of Medicine and Health, University of Exeter, 1.18 College House, St Luke’s Campus, Heavitree Road, Exeter, EX1 2LU UK; 2 Department of Surgery, University of Melbourne, Melbourne, Australia; 3 Department of Urology, Royal Melbourne Hospital, Melbourne, Australia; 4 Department of Urology, University of California San Francisco, San Francisco, USA; 5 James Buchanan Brady Urological Institute, Johns Hopkins University, Baltimore, USA; 6 Department of Urologic Surgery, Vanderbilt University, Nashville, USA; 7 Nuffield Department of Surgical Sciences, University of Oxford, Oxford, UK; 8 Division of Surgery and Interventional Medicine, University College London, London, UK; 9 Radiation Oncology, Ghent University Hospital, Ghent, Belgium

**Keywords:** Prostate cancer, Cancer treatment protocols, Patient selection, Active surveillance

## Abstract

**Background:**

Active surveillance (AS) is a management option for men diagnosed with lower risk prostate cancer. There is wide variation in all aspects of AS internationally, from patient selection to investigations and follow-up intervals, and a lack of clear evidence on the optimal approach to AS. This study aimed to provide guidance for clinicians from an international panel of prostate cancer experts.

**Methods:**

A modified Delphi approach was undertaken, utilising two rounds of online questionnaires followed by a face-to-face workshop. Participants indicated their level of agreement with statements relating to patient selection for AS via online questionnaires on a 7-point Likert scale. Factors not achieving agreement were iteratively developed between the two rounds of questionnaires. Draft statements were presented at the face-to-face workshop for discussion and consensus building.

**Results:**

12 prostate cancer experts (9 urologists, 2 academics, 1 radiation oncologist) participated in this study from a range of geographical regions (4 USA, 4 Europe, 4 Australia). Complete agreement on statements presented to the participants was 29.4% after Round One and 69.0% after Round Two. Following robust discussions at the face-to-face workshop, agreement was reached on the remaining statements. PSA, PSA density, Multiparametric MRI, and systematic biopsy (with or without targeted biopsy) were identified as minimum diagnostic tests required upon which to select patients to recommend AS as a treatment option for prostate cancer. Patient factors and clinical parameters that identified patients appropriate to potentially receive AS were agreed. Genetic and genomic testing was not recommended for use in clinical decision-making regarding AS.

**Conclusions:**

The lack of consistency in the practice of AS for men with lower risk prostate cancer between and within countries was reflected in this modified Delphi study. There are, however, areas of common practice and agreement from which clinicians practicing in the current environment can use to inform their clinical practice to achieve the best outcomes for patients.

## Background

Prostate cancer incidence globally is increasing and prostate cancer specific mortality is decreasing, in part due to an increase in the diagnosis of low-risk prostate cancer [1]. The risk of disease progression in men with localised prostate cancer at diagnosis is mainly determined by the Gleason grade, a histopathological prostate cancer grading system [2]. Patients with localised Gleason grade 3 + 3 prostate cancer have almost no risk of metastatic progression, and the risk increases with higher Gleason grades [3]. Active Surveillance (AS) is a treatment option for patients diagnosed with localised prostate cancer with low-risk of disease progression. AS involves proactive, regular monitoring to defer or avoid radical treatments that have significant associated morbidity, such as prostatectomy or radiotherapy [4]. Large single centre [5, 6] studies, multicentre [7, 8] AS cohorts, and a large randomised controlled trial of active monitoring versus radical treatment [9] have shown that the risk of disease progression and prostate cancer mortality is very low in men with low-risk disease after long-term follow-up. These findings were relatively consistent, despite each study employing different AS eligibility criteria and protocols.

A major challenge for urologists and prostate cancer multidisciplinary teams in identifying appropriate patients for AS is the accurate diagnosis and grading of prostate cancer. The traditional diagnostic pathway using prostate specific antigen (PSA) testing followed by transrectal ultrasound guided biopsy (TRUS), which published AS studies to date have largely relied upon, is known to over-detect low-grade prostate cancer and under-detect high grade prostate cancer [10]. The limitations of the traditional pathway can be seen in the rates of diagnoses in men with previous negative biopsies undergoing repeat TRUS biopsies [11] and the reclassification of 25–33% of patients on AS to higher grade disease after initial diagnosis in cohort studies [7, 8]. Multiparametric magnetic resonance imaging (mpMRI), which is a relatively new imaging modality that can be used in the pre-biopsy setting to detect lesions in the prostate and guide targeted biopsy, is a more accurate test [10, 12] whose role in AS is still being defined [13, 14]. Numerous biomarkers and genetic mutations have been identified as potentially being useful for prostate cancer risk stratification and prognostication, but the evidence base remains heterogeneous and weak [15].

Given the challenges in accurately diagnosing and classifying prostate cancer and the range of AS strategies that have been trialled to date, the wide variation in AS that currently exists in clinical practice is probably not surprising. Numerous studies have shown significant variation in all aspects of AS, including patient selection, follow-up protocols, and criteria for switching to radical treatments. This variation in AS practice has been shown to exist between institutions, regionally within countries, and in national and international guidelines [16–19]. Whilst survey data shows that the majority of clinicians perceive AS to be effective [20], the optimal approach to selecting patients for AS is still unknown. This modified Delphi study aimed to develop consensus on practical guidance for clinicians in the appropriate selection of men with lower risk prostate cancer to recommend active surveillance.

## Methods

**Fig. 1:**
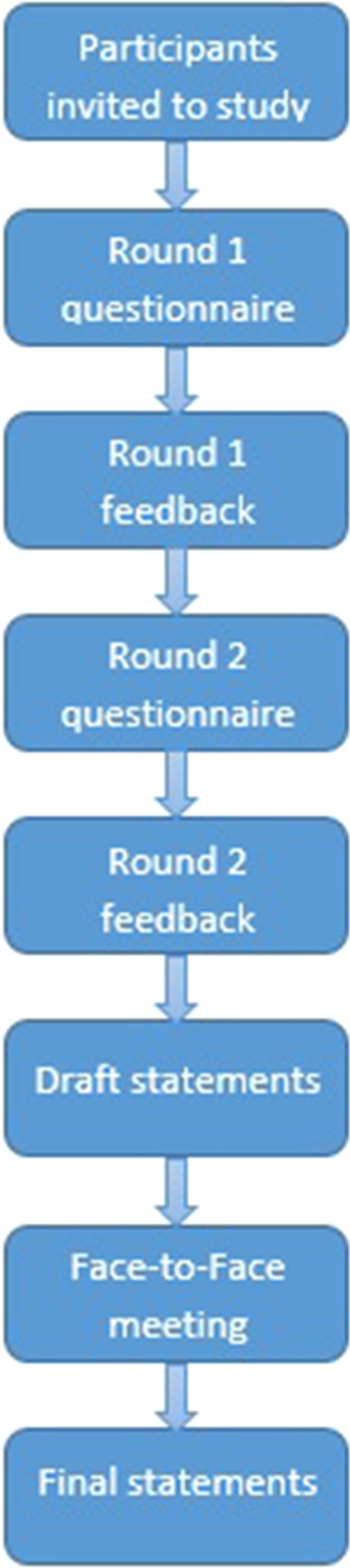
Study flow diagram

This study followed a modified Delphi method [21] to achieve consensus amongst a group of international prostate cancer experts (see Fig. 1). Participants of the 20th Asia–Pacific Prostate Cancer Conference were invited to take part in the study via email. Basic demographic information was collected from all participants. The aim of recruitment was to include at least one participant from each continent represented at the conference.

A review of the literature was undertaken to understand the current practice of AS in different healthcare systems, and previous attempts to achieve consensus in aspects of AS. Specifically, individual patient and clinical factors, and criteria for offering AS, were searched for and extracted from recently published studies of AS for prostate cancer. Any factors with at least some evidence for discrimination and patient selection for AS were included in the online questionnaires to ensure no relevant factors were missed. Key references were used to inform the development of the online questionnaires and the consensus statements.

Two rounds of anonymised questionnaires (see Additional files 1, 2: supplementary files) were delivered to participants using a secure online platform between May and July 2019. Participants were sent a link via email to complete the questionnaires, which remained open to responses for two weeks. After each round of questionnaires, the participants received a summary feedback report and were given the opportunity for comment and suggestions to feed into the iterative process of refining statements that had not yet achieved consensus.

Participants responded to individual statements about factors affecting decision-making to offer AS on a 7-point Likert scale. Agreement for each statement was defined by a mean score of 5.5 or higher on the scale. Disagreement for each statement occurred if > 33% of respondents were divergent in their views [22]. Participants were also asked to rank the relative importance of individual factors in decision-making for AS, and build their preferred criteria for AS. Free text comments were also analysed to inform the draft consensus statements for discussion and agreement.

The consensus workshop was held at the 20th Asia–Pacific Prostate Cancer Conference in Melbourne, Australia. The majority (n = 9) of Delphi study participants were in attendance. Audio-visual recording of the workshop was undertaken to document the discussions of the participants in full. The workshop was opened with a summary of the results of the online questionnaires and the areas where consensus had yet to be achieved. Draft statements were discussed and developed in the workshop, and the final criteria for AS were circulated to all participants following the workshop for agreement.

## Results

12 international prostate cancer experts participated in this modified Delphi study. Most participants were practicing urologists [9] and most were male [10]. There were 4 participants from each of the continents represented at the 20th Asia–Pacific Prostate Cancer Conference (North America, Europe, and Australia). One participant did not complete Round Two of the questionnaires, and three participants were not present for the face-to-face workshop. All participants reviewed the final AS criteria prior to publication. All eligible participants who were invited consented to participate in this study.

The initial questionnaire presented participants with 49 statements regarding individual clinical factors affecting decision-making for prostate cancer. Clear consensus was achieved on 29.4% of the statements. A ranking exercise of the relative importance of individual clinical factors showed that participants felt the Gleason score/Gleason Grade Group provided the most useful data for decision-making in relation to offering active surveillance for prostate cancer, however it was emphasised by multiple participants that the decision is multifactorial. Table 1 shows the results of an initial exercise in designing an AS protocol.

**Table 1:** Factors for selection of patients for AS achieving agreement after Round 1 questionnaire

PSA < 10 ng/mL
Clinical stage < T2
Systematic and/or Targeted biopsy approach
12 cores taken at biopsy
Gleason score 3 + 4 or lower

Utilising data obtained from Round 1 and participant feedback, a further 12 factors were added for possible inclusion in the Round 2 questionnaire. Consensus was achieved on 69% of all statements at the conclusion of this round of the study. 82% of participants also agreed that a combination of criteria should be met for recommending AS; one participant felt all criterion in any list should be met and one indicated at least one criterion should be met. After participants were presented with the Round 2 results and given the opportunity for further comment, a collection of draft consensus statements were prepared and circulated prior to the face-to-face workshop. An open meeting utilising facilitated discussion was conducted with the study participants, focusing on the areas where consensus had not yet been reached and developing the list of consensus statements into a set of criteria for recommending AS for patients with prostate cancer. The discussions were synthesised into final consensus statements, which were circulated for comment and approval after the workshop (See Table 2).

**Table 2:** Final criteria for prostate cancer patient selection for AS agreed by Delphi panel

Criteria for recommending active surveillance as a treatment option for patients with localised prostate cancer
*The following patient factors should be taken into consideration in the decision whether to recommend active surveillance to a patient as a treatment option for prostate cancer:* Medical co-morbidities Life expectancy Suitability to undergo radical treatment for prostate cancer Treatment preferences
*The results of the following tests should be considered in making a decision whether to recommend active surveillance (as a minimum):* Prostate Specific Antigen (PSA) PSA density Multiparametric MRI (mpMRI) Prostate biopsy (with a minimum of 12 cores from a systematic approach ± 2–4 targeted cores from an MRI visible lesion)
*Patients meeting all of the following criteria can be recommended active surveillance as a treatment option. Patients meeting five of the following criteria could also be considered for active surveillance:* PSA less than or equal to 10 ng/mL PSA density less than or equal to 0.15 ng/mL^2^ Clinical stage less than or equal to T1c PIRADS score less than 3 Gleason score 3 + 3/Gleason Grade Group of 1 No family history of prostate cancer
*Patients meeting the following criteria should not be recommended active surveillance as a treatment option:* Gleason score 4 + 3/Gleason Grade Group ≥ 3 Genetic and genomic testing (such as Prostarix or SNP profiles) should not be used to inform decisions about active surveillance

## Discussion

This modified Delphi study sought to address current issues surrounding the consistency and quality of care for men with prostate cancer who could potentially benefit from active surveillance as a treatment modality. The panel considered a range of issues relating to AS, including underlying principles of treatment, diagnostic information, and risk assessment. The agreed criteria outline a relatively flexible approach to offering AS, reflecting the current uncertainties and evolving evidence around the optimal use and delivery of AS.

### Key principles of active surveillance

There were a number of key principles on which the participants held clear agreement. The panel agreed that a patient with prostate cancer should be in a reasonable state of health in order to benefit from active surveillance, and that their life expectancy, medical co-morbidities, suitability for radical treatment, and treatment preferences should all be taken into consideration for management decision-making. In the context of the known limitations of the current prostate cancer diagnostic tests, with the potential for over-diagnosis and misclassification of prostate cancer, a rigorous approach to AS is critical to avoid over-treatment and the associated adverse effects for men. The panel also felt that an optimal AS follow-up protocol would be able to accurately identify disease progression to inform decisions about switching to radical treatment.

### Diagnostic information

The importance of accurate diagnostic information was discussed at length by the panel, underlying the key role that this data plays in identifying men who are potentially appropriate for AS. This is exemplified in the two cases captured in Table 3. The index of suspicion following a negative mpMRI and the need to consider biopsy in these men with some similar characteristics is clinically different, and underlines the importance of thorough investigation to make a clear diagnosis of prostate cancer. There was strong consensus on the minimum set of diagnostic tests needed to inform the decision about offering AS (see Table 2), however other available tests such as Free:Total PSA ratio and the number of positive cores were more contentious. Multiparametric MRI was felt to be of high importance, and is increasingly being used as a diagnostic test for prostate cancer; however pre-biopsy mpMRI has not yet been recommended in many national level guidelines [17, 23].

**Table 3:** Clinical scenarios of two similar patients with different prostate cancer risk

	Patient 1	Patient 2
Age	50	50
PSA	10	10
PIRADS 2.1	1	1
Free:Total PSA	Low	High
Family history of prostate cancer	Negative	Negative
Prostate volume	20 cc	120 cc

The number of positive cores and positive core length can be influenced by the location of the biopsy sampling and the total number of cores taken, and the relative importance of these factors was not agreed upon by the panel. Most prostate cancer guidelines do not include the number of positive cores or the percentage of cancer per core in prostate cancer risk definitions, although many individual centres still include these measures in their AS eligibility criteria [17, 18]. The panel generally felt that Transrectal Ultrasound-guided biopsy (TRUS) alone was not sufficiently accurate to be used as the basis for histopathological assessment of a prostate cancer, and ideally a Transperineal (TP) biopsy approach was followed. This reflects the general trend towards increasing use of TP biopsy due to its lower adverse effect profile and ability to access all areas of the prostate [24]. The need for a confirmatory biopsy in tertiary centre referrals was also debated, with no clear opinion reached; however the importance of concordance between mpMRI and biopsy findings was felt to be vital in reassuring the clinicians and the patient that an accurate diagnosis has been made.

### Prognostication of localised prostate cancer

A clear challenge for clinicians treating men with localised prostate cancer at the present time is accurately assessing the risk of disease progression in men with Gleason score 3 + 4/Gleason Grade Group 2, and recommending the appropriate treatment. The risks of morbidity from radical treatment versus the risks of disease progression on AS are often finely balanced. This was a particularly contentious issue for the panel, and no consensus was reached. The agreed statements in Table 2 could be considered conservative in their approach, but the majority of panel members were more comfortable with not recommending AS as the optimal treatment option to a patient given there is currently no reliable way of distinguishing lethal from non-lethal prostate cancer for these men.

### Strengths and limitations

This modified Delphi study was conducted in a methodologically rigorous manner. A diverse panel of international prostate cancer experts was assembled, with an optimal number of participants who engaged throughout the whole process. Agreement was achieved on the consensus statements through robust discussion and iterative work over two rounds of questionnaires, followed by a face-to-face workshop to refine the criteria [25]. The panel has developed a set of practical recommendations that take into consideration the latest evidence in the field. The role of certain tests, including number of positive cores and Free:Total PSA ratio, in patient selection for AS was not agreed by the panel, although this could be considered to be a reflection of the current state of the evidence in these areas [15].

## Conclusions

Active surveillance is an appropriate treatment option for men with localised, low-risk prostate cancer. It can be utilised to achieve good outcomes for patients and avoid overtreatment. It is vital that complete and accurate diagnostic information is obtained to ensure that the correct patients are recommended to undergo AS, and a patient’s suitability and treatment preferences are factored into the shared decision-making process. The findings of cohort studies of patients receiving AS that are currently on-going will help to fill some of the evidence gaps around patient selection and follow-up protocols that exist today.

## Supplementary Information

**Additional file 1: **Round 1 online questionnaire.

**Additional file 2: **Round 2 online questionnaire.

## Data Availability

The datasets generated and/or analysed during the current study are not publicly available as consent was not sought from participants to make data publically available. Data may be available from the corresponding author on reasonable request.

## Abbreviations

AS: Active surveillance
mpMRI: Multiparametric magnetic resonance imaging
PIRADS: Prostate image-reporting and data system
PSA: Prostate specific antigen
TP: Transperineal
TRUS: Transrectal ultrasound guided biopsy
USA: United States of America
