# Supplementary material for: A modified Delphi study to develop a practical guide for selecting patients with prostate cancer for active surveillance
Source: BMC Urol. 2021 Feb 4;21:18. doi: 10.1186/s12894-021-00789-5 (PMC7863517; doi:10.1186/s12894-021-00789-5)
Supplement: Supplementary file 1 — Additional file 1: Round 1 online questionnaire. [file 12894_2021_789_MOESM1_ESM.docx]

Round 1 questionnaire

**Survey questions**

Individual factors

Please indicate your level of agreement from strongly agree (7) to strongly disagree (1) about whether each of these individual factors are important in the decision making for recommending active surveillance as a treatment option to a patient with prostate cancer.

*Prostate specific antigen (PSA)*

Patients with prostate cancer and a PSA score of 10 or less at diagnosis should be considered for active surveillance

Strongly disagree Strongly agree

1 2 3 4 5 6 7

Patients with prostate cancer and a PSA score of 15 or less at diagnosis should be considered for active surveillance

Strongly disagree Strongly agree

1 2 3 4 5 6 7

Patients with prostate cancer and a PSA score of 20 or less at diagnosis should be considered for active surveillance

Strongly disagree Strongly agree

1 2 3 4 5 6 7

Patients with prostate cancer and a PSA density of 0.15 or less at diagnosis should be considered for active surveillance

Strongly disagree Strongly agree

1 2 3 4 5 6 7

Patients with prostate cancer and a PSA density of 0.20 or less at diagnosis should be considered for active surveillance

Strongly disagree Strongly agree

1 2 3 4 5 6 7

*Multiparametric magnetic resonance imaging (mpMRI)*

mpMRI should be performed pre-biopsy as part of the diagnostic work-up for patients with prostate cancer

Strongly disagree Strongly agree

1 2 3 4 5 6 7

If not already performed, mpMRI should be done after biopsy to confirm the diagnosis for patients with low risk prostate cancer prior to being offered active surveillance

Strongly disagree Strongly agree

1 2 3 4 5 6 7

Patients with prostate cancer and a PIRADs score of 3 should be considered for active surveillance

Strongly disagree Strongly agree

1 2 3 4 5 6 7

Patients with prostate cancer and a PIRADs score of 4 should be considered for active surveillance

Strongly disagree Strongly agree

1 2 3 4 5 6 7

*Biopsy*

Targeted biopsy should be performed as part of the diagnostic work-up to inform the decision about whether to offer active surveillance to a patient with prostate cancer

Strongly disagree Strongly agree

1 2 3 4 5 6 7

Systematic biopsy should be performed as part of the diagnostic work-up to inform the decision about whether to offer active surveillance to a patient with prostate cancer

Strongly disagree Strongly agree

1 2 3 4 5 6 7

Template biopsy should be performed as part of the diagnostic work-up to inform the decision about whether to offer active surveillance to a patient with prostate cancer

Strongly disagree Strongly agree

1 2 3 4 5 6 7

Anterior zone biopsy sampling should be performed in patients with prostate cancer before offering active surveillance

Strongly disagree Strongly agree

1 2 3 4 5 6 7

A minimum of 8 cores should be taken at prostate biopsy to inform decisions about offering active surveillance to patients with prostate cancer

Strongly disagree Strongly agree

1 2 3 4 5 6 7

A minimum of 10 cores should be taken at prostate biopsy to inform decisions about offering active surveillance to patients with prostate cancer

Strongly disagree Strongly agree

1 2 3 4 5 6 7

A minimum of 12 cores should be taken at prostate biopsy to inform decisions about offering active surveillance to patients with prostate cancer

Strongly disagree Strongly agree

1 2 3 4 5 6 7

Minimum number of cores taken at prostate biopsy to inform decisions about offering active surveillance to patients with prostate cancer should be decided by the individual clinician

Strongly disagree Strongly agree

1 2 3 4 5 6 7

The maximum number of positive cores from prostate biopsy that is acceptable for offering a patient with prostate cancer to undergo active surveillance is 2

Strongly disagree Strongly agree

1 2 3 4 5 6 7

The maximum number of positive cores from prostate biopsy that is acceptable for offering a patient with prostate cancer to undergo active surveillance is 3

Strongly disagree Strongly agree

1 2 3 4 5 6 7

The maximum number of positive cores from prostate biopsy that is acceptable for offering a patient with prostate cancer to undergo active surveillance is 4

Strongly disagree Strongly agree

1 2 3 4 5 6 7

The maximum proportion of positive cores from prostate biopsy that is acceptable for offering a patient with prostate cancer to undergo active surveillance is 20%

Strongly disagree Strongly agree

1 2 3 4 5 6 7

The maximum proportion of positive cores from prostate biopsy that is acceptable for offering a patient with prostate cancer to undergo active surveillance is 33%

Strongly disagree Strongly agree

1 2 3 4 5 6 7

The maximum proportion of positive cores from prostate biopsy that is acceptable for offering a patient with prostate cancer to undergo active surveillance is 50%

Strongly disagree Strongly agree

1 2 3 4 5 6 7

The maximum percentage of any biopsy core being positive from prostate biopsy that is acceptable for offering a patient with prostate cancer to undergo active surveillance is 30%

Strongly disagree Strongly agree

1 2 3 4 5 6 7

The maximum percentage of any biopsy core being positive from prostate biopsy that is acceptable for offering a patient with prostate cancer to undergo active surveillance is 50%

Strongly disagree Strongly agree

1 2 3 4 5 6 7

The maximum length of any biopsy core being positive from prostate biopsy that is acceptable for offering a patient with prostate cancer to undergo active surveillance is 4mm

Strongly disagree Strongly agree

1 2 3 4 5 6 7

The maximum length of any biopsy core being positive from prostate biopsy that is acceptable for offering a patient with prostate cancer to undergo active surveillance is 6mm

Strongly disagree Strongly agree

1 2 3 4 5 6 7

*Clinical stage*

Patient with prostate cancer and a clinical stage of T1c should be considered for active surveillance

Strongly disagree Strongly agree

1 2 3 4 5 6 7

Patient with prostate cancer and a clinical stage of T2a or less should be considered for active surveillance

Strongly disagree Strongly agree

1 2 3 4 5 6 7

Patient with prostate cancer and a clinical stage of T2b or less should be considered for active surveillance

Strongly disagree Strongly agree

1 2 3 4 5 6 7

Patient with prostate cancer and a clinical stage of T2 or less should be considered for active surveillance

Strongly disagree Strongly agree

1 2 3 4 5 6 7

Patients with multifocal prostate cancer should be considered for active surveillance

Strongly disagree Strongly agree

1 2 3 4 5 6 7

*Gleason score*

Patients with prostate cancer and a Gleason score less than or equal to 3+3 should be considered for active surveillance

Strongly disagree Strongly agree

1 2 3 4 5 6 7

Patients with prostate cancer and a Gleason score less than or equal to 3+4 should be considered for active surveillance

Strongly disagree Strongly agree

1 2 3 4 5 6 7

Patients with prostate cancer and a Gleason score less than or equal to 4+3 should be considered for active surveillance

Strongly disagree Strongly agree

1 2 3 4 5 6 7

*Gleason grade*

Patients with prostate cancer and a Gleason grade group of 1 should be considered for active surveillance

Strongly disagree Strongly agree

1 2 3 4 5 6 7

Patients with prostate cancer and a Gleason grade group of 2 or less should be considered for active surveillance

Strongly disagree Strongly agree

1 2 3 4 5 6 7

Patients with prostate cancer and a Gleason grade group of 3 or less should be considered for active surveillance

Strongly disagree Strongly agree

1 2 3 4 5 6 7

*Family history*

Patients with prostate cancer and a family history of prostate cancer should be considered for active surveillance

Strongly disagree Strongly agree

1 2 3 4 5 6 7

Patients with prostate cancer and a BRCA1/2 mutation should be considered for active surveillance

Strongly disagree Strongly agree

1 2 3 4 5 6 7

Patients with prostate cancer and lynch syndrome should be considered for active surveillance

Strongly disagree Strongly agree

1 2 3 4 5 6 7

Patients with prostate cancer and a MMR germline mutation should be considered for active surveillance

Strongly disagree Strongly agree

1 2 3 4 5 6 7

Patients with prostate cancer and an ATM germline mutation should be considered for active surveillance

Strongly disagree Strongly agree

1 2 3 4 5 6 7

*Novel biomarkers and polygenic risk scores*

The Prostate Health Index should be used to inform decisions about offering active surveillance to men with prostate cancer

Strongly disagree Strongly agree

1 2 3 4 5 6 7

PSA3 should be used to inform decisions about offering active surveillance to men with prostate cancer

Strongly disagree Strongly agree

1 2 3 4 5 6 7

The 4K panel should be used to inform decisions about offering active surveillance to men with prostate cancer

Strongly disagree Strongly agree

1 2 3 4 5 6 7

The Decipher test should be used to inform decisions about offering active surveillance to men with prostate cancer

Strongly disagree Strongly agree

1 2 3 4 5 6 7

The Prolaris score should be used to inform decisions about offering active surveillance to men with prostate cancer

Strongly disagree Strongly agree

1 2 3 4 5 6 7

The Prostarix test should be used to inform decisions about offering active surveillance to men with prostate cancer

Strongly disagree Strongly agree

1 2 3 4 5 6 7

Prostate cancer risk single nucleotide polymorphisms (SNP) risk profile scores should be used to inform decisions about offering active surveillance to men with prostate cancer

Strongly disagree Strongly agree

1 2 3 4 5 6 7

*Other factors*

If you consider any other factors to be important in the decision to offer a patient with prostate cancer active surveillance as a treatment option, please enter them in the space below

[FREE TEXT]

Ranking exercise

Below is a list of individual patient factors that may be considered in the decision to offer or recommend active surveillance as a treatment option for prostate cancer. Please rank them in order from most important (1) to least important (18) in your clinical opinion.

PSA

Targeted biopsy result

Systemic biopsy result

Template biopsy result

Fusion biopsy result

Clinical stage

Number of cores

Number of positive cores

Proportion of positive cores

Percentage of core involvement

Core length

Clinical stage

Gleason core

Gleason Grade Group

Family history

Germline mutations status (eg BRCA, Lynch Syndrome)

PIRADS v2 score

Polygenic risk scores (eg PHI, SNP profiles)

Active Surveillance protocol

Based on your understanding of the current evidence and best practice in decision-making for active surveillance in patients with prostate cancer, please indicate which combination of the following factors should be taken into consideration when making a recommendation for active surveillance as a treatment option. Please select as many or as few factors as you feel are important in this area

*PSA*

PSA < 10

PSA < 15

PSA <20

*PSA density*

PSA density < 0.15

PSA density < 0.20

*Clinical stage*

T1c or less

T2a or less

T2b or less

T2c or less

mpMRI

PIRADS 3

PIRADS 4

*Biopsy*

Systematic biopsy performed

Targeted biopsy performed

Template biopsy performed

Minimum 8 cores taken at biopsy

Minimum 10 cores taken at biopsy

Minimum 12 cores taken at biopsy

Maximum of two positive cores

Maximum of three positive cores

Maximum of four positive cores

Up to 20% of cores positive

Up to 33% of cores positive

Up to 50% of cores positive

Maximum positive core length is 4mm

Maximum positive core length is 6mm

*Pathology*

Gleason score 3+3 or lower

Gleason score 3+4 or lower

Gleason score 4+3 or lower

Gleason Grade Group 1

Gleason Grade Group 2 or lower

Gleason Grade Group 3 or lower

*Family history*

Absence of family history of prostate cancer

Absence of any inherited germline mutations (eg BRCA, Lynch syndrome)

Polygenic risk score – [FREE TEXT = PLEASE SPECIFY]
