# Supplementary material for: A modified Delphi study to develop a practical guide for selecting patients with prostate cancer for active surveillance
Source: BMC Urol. 2021 Feb 4;21:18. doi: 10.1186/s12894-021-00789-5 (PMC7863517; doi:10.1186/s12894-021-00789-5)
Supplement: Supplementary file 2 — Additional file 2: Round 2 online questionnaire. [file 12894_2021_789_MOESM2_ESM.docx]

Round 2 questionnaire

Individual factors

Please indicate your level of agreement from strongly agree (7) to strongly disagree (1) about whether each of these individual factors are important in the decision making for recommending active surveillance as a treatment option to a patient with prostate cancer.

*PSA density*

PSA density should be measured to inform the decision about whether to offer active surveillance to a patient with prostate cancer

Strongly disagree Strongly agree

1 2 3 4 5 6 7

*Free/total PSA ratio*

Patients with prostate cancer and a Free/total PSA ratio of 0.10 or less at diagnosis should be considered for active surveillance

Strongly disagree Strongly agree

1 2 3 4 5 6 7

Patients with prostate cancer and a Free/total PSA ratio of 0.20 or less at diagnosis should be considered for active surveillance

Strongly disagree Strongly agree

1 2 3 4 5 6 7

Patients with prostate cancer and a Free/total PSA ratio of 0.25 or less at diagnosis should be considered for active surveillance

Strongly disagree Strongly agree

1 2 3 4 5 6 7

*Biopsy*

Prostate biopsy should use a transrectal approach as part of the diagnostic work-up to inform the decision about whether to offer active surveillance to a patient with prostate cancer

Strongly disagree Strongly agree

1 2 3 4 5 6 7

Prostate biopsy should use a transperineal approach as part of the diagnostic work-up to inform the decision about whether to offer active surveillance to a patient with prostate cancer

Strongly disagree Strongly agree

1 2 3 4 5 6 7

It does not matter whether biopsy uses a transrectal or transperineal approach as part of the diagnostic work-up to inform the decision about whether to offer active surveillance to a patient with prostate cancer

Strongly disagree Strongly agree

1 2 3 4 5 6 7

*Patient factors*

The patient’s age is important in informing the decision about whether to offer active surveillance to a patient with prostate cancer

Strongly disagree Strongly agree

1 2 3 4 5 6 7

The patient’s co-morbidities are important in informing the decision about whether to offer active surveillance to a patient with prostate cancer

Strongly disagree Strongly agree

1 2 3 4 5 6 7

The patient’s life expectancy is important in informing the decision about whether to offer active surveillance to a patient with prostate cancer

Strongly disagree Strongly agree

1 2 3 4 5 6 7

The patient’s treatment preferences are important in informing the decision about whether to offer active surveillance to a patient with prostate cancer

Strongly disagree Strongly agree

1 2 3 4 5 6 7

The patient’s suitability to undergo radical treatment (i.e. prostatectomy, radiotherapy) is important in informing the decision about whether to offer active surveillance to a patient with prostate cancer

Strongly disagree Strongly agree

1 2 3 4 5 6 7

*Other factors*

If you consider any other factors to be important in the decision to offer a patient with prostate cancer active surveillance as a treatment option, please enter them in the space below

[FREE TEXT]

Ranking exercise

Below is a list of individual patient factors that may be considered in the decision to offer or recommend active surveillance as a treatment option for prostate cancer, based on Round 1 of this study. Please indicate the three most important factors for offering active surveillance in rank order (1 = most important)

PSA

Clinical stage

Gleason score

Gleason Grade Group

PIRADS v2 score

Patient age

Patient treatment preferences

Active Surveillance protocol

Based on Round 1 of this study, the following factors were identified as important criteria for recommending active surveillance to patients with prostate cancer.

PSA < 10

PSA density <0.15

Clinical stage T1c or less

PIRADs $\leq$ 3

Systematic or targeted biopsy performed, with a minimum of 12 cores taken

Gleason score 3+4 / Gleason Grade group 2 or lower

Please indicate whether there are any other important factors not included above

[Free text]

Please provide any comments you have on the above criteria

[Free text]

How should the criteria above be applied in the decision-making for recommending active surveillance for men with prostate cancer?

All criteria must be met

At least two criteria must be met

At least one criteria must be met

A combination of criteria – please specify [Free text]
